# Molecular Targeted Therapies of Aggressive Thyroid Cancer

**DOI:** 10.3389/fendo.2015.00176

**Published:** 2015-11-20

**Authors:** Silvia Martina Ferrari, Poupak Fallahi, Ugo Politti, Gabriele Materazzi, Enke Baldini, Salvatore Ulisse, Paolo Miccoli, Alessandro Antonelli

**Affiliations:** ^1^Department of Clinical and Experimental Medicine, University of Pisa, Pisa, Italy; ^2^Department of Surgical, Medical, Molecular Pathology and Critical Area, University of Pisa, Pisa, Italy; ^3^Department of Experimental Medicine, Sapienza University of Rome, Rome, Italy

**Keywords:** papillary thyroid cancer, follicular thyroid cancer, medullary thyroid cancer, dedifferentiated thyroid cancer, sorafenib, vandetanib, cabozantinib, lenvantinib

## Abstract

Differentiated thyroid carcinomas (DTCs) that arise from follicular cells account >90% of thyroid cancer (TC) [papillary thyroid cancer (PTC) 90%, follicular thyroid cancer (FTC) 10%], while medullary thyroid cancer (MTC) accounts <5%. Complete total thyroidectomy is the treatment of choice for PTC, FTC, and MTC. Radioiodine is routinely recommended in high-risk patients and considered in intermediate risk DTC patients. DTC cancer cells, during tumor progression, may lose the iodide uptake ability, becoming resistant to radioiodine, with a significant worsening of the prognosis. The lack of specific and effective drugs for aggressive and metastatic DTC and MTC leads to additional efforts toward the development of new drugs. Several genetic alterations in different molecular pathways in TC have been shown in the past few decades, associated with TC development and progression. Rearranged during transfection (RET)/PTC gene rearrangements, RET mutations, BRAF mutations, RAS mutations, and vascular endothelial growth factor receptor 2 angiogenesis pathways are some of the known pathways determinant in the development of TC. Tyrosine kinase inhibitors (TKIs) are small organic compounds inhibiting tyrosine kinases auto-phosphorylation and activation, most of them are multikinase inhibitors. TKIs act on the aforementioned molecular pathways involved in growth, angiogenesis, local, and distant spread of TC. TKIs are emerging as new therapies of aggressive TC, including DTC, MTC, and anaplastic thyroid cancer, being capable of inducing clinical responses and stabilization of disease. Vandetanib and cabozantinib have been approved for the treatment of MTC, while sorafenib and lenvatinib for DTC refractory to radioiodine. These drugs prolong median progression-free survival, but until now no significant increase has been observed on overall survival; side effects are common. New efforts are made to find new more effective and safe compounds and to personalize the therapy in each TC patient.

## Introduction

The incidence of papillary thyroid cancer (PTC) is increasing (1), and it is the predominant endocrine carcinoma (seventh most common cause of new cancer in the US women).

The increasing diagnosis of small thyroid cancers (TCs) is related to the use of neck ultrasonography and fine needle aspiration (FNA) of thyroid nodules, and also to ionizing radiations (2–4% of subjects exposed to radiation during childhood show TC) (2), the exposure to fall-out of nuclear explosions or accidents (3), low doses of radiation exposure (4), and the exposure to iodine deficient areas, where a higher frequency of follicular thyroid cancer (FTC) has been observed (5). Furthermore, autoimmune thyroiditis has been shown to be a risk factor for PTCs (6, 7), and new risk factors are emerging (8, 9).

According to histopathological criteria: (1) differentiated thyroid carcinomas (DTCs) that arise from follicular cells account >90% of TC and are subdivided into PTC (~90%) or FTC (~10%); (2) medullary thyroid cancer (MTC) accounts <5% of TC [sporadic (75%); or hereditary forms (25%) that include familial MTC, multiple endocrine neoplasia type 2 (MEN) 2A, and MEN 2B (10, 11); and (3) anaplastic thyroid cancer (ATC) accounts for <2%.

Complete total thyroidectomy is the treatment of choice for PTC, FTC, and MTC (12). Radioactive iodine (RAI – 131I) should be routinely recommended in high-risk patients and considered in intermediate risk DTC patients (13).

Patients with PTC and FTC (after surgery, and eventually RAI) are followed by neck ultrasonography and the determination of basal or thyroid-stimulating hormone (TSH)-stimulated thyroglobulin (Tg) (14–16).

More than 90% of patients with DTC have a normal life expectancy (17). The prevalence of TC deaths per year (in relation to the number of new cases) is decreased from 15 to 5% in the past few decades (10), in contrast with the increasing incidence of the disease.

At the diagnosis, ~5% of DTC patients have distant metastasis (50% lungs, 25% bones, 20% lungs and bones, and 5% other sites); 10–15% of patients show recurrences in the follow-up (in the thyroid bed, or lymph nodes), with a survival reduction (from 70 to 50%, at 10 years) (18, 19). Approximately one-third of cancer-related deaths are associated with neck lesions alone (20).

DTC cancer cells, during tumor progression, may lose the iodide uptake ability, becoming resistant to RAI, with a significant worsening of the prognosis (17–19).

Poorly differentiated thyroid carcinomas (PDTCs) are a subset of thyroid tumors, considered intermediate between DTC and ATC, and they are more aggressive than DTC, but less than ATC (21, 22).

When tumor progresses, tumor cells lose the iodide uptake ability and cancer becomes resistant to the traditional therapeutic strategies (23).

In thyrocytes, the sodium/iodine symporter (NIS) (24, 25) regulates the iodide trapping, that is a TSH-regulated mechanism, at the basolateral surface of the cell with an energy-dependent transport, while at the apical surface [where a role has been suggested for the Pendred syndrome (PDS) gene], iodide trapping is regulated by a passive transport. At the apical surface, the iodide is organified by thyroperoxidase (TPO) and conjugated to tyrosine residues on Tg. In primary and metastatic TC, a decrease in NIS transcripts has been shown with respect to normal tissues, that is more evident in metastases unable to uptake 131I with respect to primary cancers, or metastases able to trap it. Also Tg, TPO, and PDS gene expressions are decreased in TC with respect to normal tissues, overall in metastases with no 131I uptake. These data suggest that not only the genetic control over NIS transcription occurs but also other mechanisms might be involved in this failure to trap 131I (26). A demonstrable 131I uptake by TC requires not only a functional and correctly located NIS but also the full machinery responsible for iodide retention in the cell. Gene therapy studies (where the NIS gene was introduced in non-TC cells to promote 131I uptake and induce cytotoxicity) confirm this hypothesis.

These studies showed that although an efficient iodine uptake was permitted by NIS delivery in the target cells, therapeutic effects were observed only with doses of radioiodine beyond the ranges used in human beings (26).

In the management of TC patients with high serum Tg levels and negative 131I WBS, the effectiveness of fludeoxyglucose-positron emission tomography (FDG-PET) has been demonstrated (27).

The ability of TC to trap fludeoxyglucose is consistent with studies that have shown a high glucose consumption in TC, accompanied by an increase in its transmembrane transport due to GLUT-1 overexpression, overall in more aggressive TC histotypes, and in the presence of metastases.

*In vivo* studies have also shown that the FDG-PET scan became more sensitive after administering recombinant human TSH, revealing lesions not observed in conditions of TSH suppression, and inducing changes in the surgical management of these patients that ameliorate their outcome (27).

In PTC, rearranged during transfection (RET)/PTC rearrangements, RAS and BRAF mutations (28), and β-catenin mutations (29) underlie the loss of iodide uptake ability.

Radiotherapy and chemotherapy (doxorubicin) are of limited efficacy in the treatment of dedifferentiated TC (30).

### Molecular Pathways Implicated in Thyroid Cancer

Various molecular pathways are implicated in the pathogenesis of TC.

Rearranged during transfection is a proto-oncogene encoding a transmembrane protein harboring a tyrosine kinase (TK) (31). Rearrangements and mutations able to activate RET have been identified in different human cancers (32). In ~40% of adult sporadic PTC, RET/PTC rearrangements are present (33); RET/PTC1 and RET/PTC3 are the most frequent, and they are commonly identified in microcarcinomas and, also, in benign thyroid lesions. For these reasons, it has been hypothesized that RET/PTC are determinant for tumor initiation, but not progression (34, 35).

The BRAF kinase belongs to the RAF family proteins (35). After activation by RAS binding and protein recruitment to the cell membrane, these kinases phosphorylate and activate MEK, that in turn activates ERK and the effectors of the MAPK cascade (33).

Valine to glutamate substitution at residue 600 (V600E) is present in ~45% PTC and rarely in FTC and is correlated with the tumor aggressiveness at presentation, with the risk of tumor recurrence, and with the loss of iodide uptake (33, 36). Other activating BRAF mutations have been evidenced in other positions (for instance, 599 and 601), but their prevalence is definitely lower than in 600 (36).

Recently, it has been demostrated that BRAF mutation in PTC is associated with a more aggressive behavior, loss of differentiation state, and decreased expression of iodide-metabolizing (37) and NIS genes (38).

It has been shown that the BRAF V600E oncogene induces TGF-Beta secretion that represses NIS expression and increases malignancy in TC (39). Furthermore, targeted BRAF V600E expression in thyrocytes of transgenic mice results in TC, which undergo dedifferentiation (40).

K-RAS, N-RAS, and H-RAS belong to the RAS gene family, encoding intracellular G-proteins that take part in the activation of intracellular signaling pathways. RAS mutations are present in ~10% of PTCs and ~40–50% of FTCs. RAS mutations have been strictly linked to a more aggressive TC behavior (41).

PAX8/PPARγ rearrangements (42) are present in ~30–40% of conventional FTC and ~5% of oncocytic carcinomas, and their presence is often associated with a good prognosis. Tumors having PAX8/PPARγ rearrangements commonly do not present RAS mutations, and this situation suggests that there are two independent pathways linked to PAX8/PPARγ translocations or RAS mutations that support the FTC development (42). PAX8/PPARγ rearrangements are evidenced in 2–10% of follicular adenomas, or in the follicular variant of PTC (43). PAX8/PPARγ translocations have been shown in 0–1% of PTC.

Increased angiogenesis correlates with a more aggressive TC behavior, and the expression of angiogenesis inhibitors or stimulators [VEGF/VEGF receptor (VEGFR), epidermal growth factor (EGF)/EGFR, platelet-derived growth factor (PDGF)/PDGFR, fibroblast growth factor (FGF)/FGFR, and hepatocyte growth factor (HGF)/c-Met] in TC is associated with clinical features of the disease (44). VEGF is more expressed (such as its main receptor VEGFR-2) in DTC, and it takes part in neoplastic progression and aggressiveness. The dispensing of drugs targeting VEGF pathway is actually a therapeutic option for TC patients (45).

VEGF A-C, placental growth factor (PlGF) and PDGF A-D belong to the VEGF gene family (46). VEGF mediates endothelial cell adhesion and migration on extracellular matrix, and this is why it is associated with an increased aggressiveness, growth, and distant spread of several tumors, including TC (46, 47).

In most DTCs, VEGF is overexpressed and its main receptor VEGFR-2 is upregulated (48).

The overexpression of angiopoietin-2 and VEGF in TC progression and a strong association between tumor size and high levels of VEGF and angiopoietin-2 were evidenced. Moreover, an increased expression of VEGF-C in lymph node invasive thyroid tumors was shown, such as a decrease of the angioinhibitory factor thrombospondin-1 in thyroid malignancies capable of hematic spread. These data support the idea that angiogenesis factors are involved in neoplastic growth, progression, and aggressiveness in human TC (49).

For this reason, the systemic administration of antiangiogenic drugs targeting components of the VEGF-A-VEGF signal transduction pathway has become a therapeutic option for patients with TC (36).

The EGFR cell-surface protein (ErbB-1; HER1 in human beings) is a receptor for the EGF-family (50). This protein belongs to a subfamily of four related receptor TKs (the ErbB-1, -2, -3, and -4). The upregulation or the overactivity of EGFR, caused by mutations, has been correlated with different cancers, for example, anal and lung cancers (51), and glioblastoma multiforme, and in the last one, the most observed mutation is EGFRvIII (52). Approximately 30% of all epithelial cancers have mutations, misregulations, or amplifications of EGFR or other family members, in fact EGFR participates in the tumor progression and invasion in TC, and it is overexpressed in ATC (53).

EGFR is determinant in TC growth and spread, and it is strongly expressed in aggressive TC. Its mutations contribute to RET activation in TC (54, 55), while RET/PTC1 and RET/PTC3 upregulate EGFR expression, with a magnitude of induction similar to that for TSH (54).

The expression of EGFR1 protein is significantly upregulated in PDTC and ATC, and absent or slight in normal thyroid gland and in papillary DTC, suggesting that upregulation of EGFR1 expression may be a molecular marker of dedifferentiation in thyroid epithelial carcinomas (56).

High expression of EGFR is associated with lymph node metastasis in PTC and plays a role in the progression of TC (53, 57, 58).

It has been also reported that a patient with metastatic PDTC with an EGFR mutation responded to the treatment with the selective EGFR tyrosine kinase inhibitor (TKI) erlotinib (59).

NH2-terminal lysine residues are acetylated on histones, and this way is the principal one that controls the cellular differentiation and biological behavior of tumoral cells. The increasing rate of the gene transcription is due to a more open chromatin configuration, while a closed chromosomal configuration leads to transcriptional repression (48). In some cancer cells, the activity of histone acetyltransferase or histone deacetylase (HDAC) has been dysregulated (60). Vorinostat, depsipeptide, valproic acid, belinostat, and panobinostat have an antineoplastic effect on TC cells (61).

### Thyroid Cancer: Targeted Therapy

#### RET Pathway

One of the most explored chemical template is the pyrazolo[3,4-d]pyrimidine (PP) heterocyclic core, shown to be a useful scaffold for the obtainment of effective TKIs (Table 1). Actually, derivatives belonging to this structural class show a large spectrum of activity. Different PP compounds act as: (a) Abl inhibitors and antiproliferative agents against human leukemia cell lines; (b) Src kinase inhibitors in neuroblastoma, medulloblastoma, and osteosarcoma; (c) phospholipase D inhibitors in different neoplasias; and (d) urokinase plasminogen activator inhibitors, in breast cancer.

**Table 1:** **Principal involved pathways and relative targeted therapies in thyroid cancer**.

Involved pathways	Drugs	Thyroid cancer	Responses				Authors
			PR	SD	PD	PFS (months)	
*Raf*	Sorafenib	30 DeTC	23.3%	53.3%	7%	21	Gupta-Abramson et al. (62)
	Sorafenib	41 DeTC	15%	56%		15	Kloos et al. (63)
	Sorafenib	207 DeTC				10.8	Brose et al. (64)
*VEGF*	Vandetanib	30 MTC	20%	53%	3%	27.9	Wells et al. (65)
	Vandetanib	231 MTC	45%	42%			Wells et al. (66)
	Vandetanib	145 DeTC	8%	57%		11.1	Leboulleux et al. (67)
	Motesanib	93 DeTC	14%	35%	8%	40 weeks	Sherman et al. (68)
	Motesanib	93 DeTC		48% MTC		40 weeks DeTC	Bass et al. (69)
		91 MTC				48 weeks MTC	
	Axitinib	49 DeTC	30%	38%	7%	18.1	Cohen et al. (70)
		11 MTC					
	Axitinib	52 MTC	35%	35%		16	Locati et al. (71)
	Sunitinib	7 MTC	28% PR + 3% CR	46%	17%	12.8	Carr et al. (72)
		28 DeTC					
	Sunitinib	11 DeTC	18% PR + 9% CR	45%	27%	11.5	Dìez et al. (73)
	Cabozantinib	37 MTC	29%				Kurzrock et al. (74)
	Cabozantinib	15 DeTC	53%	40%			Cabanillas et al. (75)
	Pazopanib	37 DeTC	49%		73%	11.7	Bible et al. (76)
	Lenvatinib	58 DeTC	50%	28%	5%	12.6	Cabanillas et al. (77)
	Lenvatinib	261 DeTC	63 PR% + 2%			18.3	Schlumberger et al. (78)
*Vascular disrupting*	Combretastatin	18 ATC		33%		7.4 weeks	Cooney et al. (79)
*EGFR*	Gefitinib	27 DeTC		12%		3.7	Pennell et al. (80)
*Histone deacetylase*	Vorinostat	16 DTC	0%	0%	36%		Woyach et al. (81)
		3 MTC					

*ATC, anaplastic thyroid cancer; DeTC, dedifferentiated thyroid cancer; MTC, medullary thyroid cancer; PR, partial response; PD, progressive disease; PFS, progression-free survival; SD, stable disease*.

Recently, CLM3, [(R)-1-phenethyl-N-(1-phenylethyl)-1H-pyrazolo[3,4-d]pyrimidin-4-amine], has been shown to inhibit RET-TK, BRAF, VEGFR-2, and EGFR and to exert antiangiogenic activity. In human TC cell lines, CLM3 showed antiproliferative and proapoptotic effects and also an antiangiogenic effect (82–84).

It has been also shown that CLM3 and CLM29 (another pyrazolo[3,4-d]pyrimidine, inhibiting RET, EGFR, and VEGFR, with antiangiogenic activity) (82) inhibited the migration of papillary dedifferentiated thyroid cancer (DePTC) cells. Moreover, CLM3 (85), CLM29 (82), and CLM94 (86) have been demonstrated to exert antineoplastic activity in primary ATC cells. CLM3 and CLM29 had an inhibitory effect independently from the presence of V600EBRAF mutation both in DePTC and in ATC. A newly produced DePTC cell line (AL), with V600EBRAF mutation, was able to grow in nu/nu mice when inoculated sc. CLM3 and CLM29 increased TSP-1 expression in the AL cell line. The antineoplastic activity of CLM3 and CLM29 could depend on the antiproliferative effect linked to apoptosis in the tumoral cells and the inhibition of migration and the neoplastic neovascularization. In fact, a significant decrease of microvessels was observed, *in vivo*, in the CLM3-treated tumors.

More recently, CLM29 was tested both in primary MTC (pMTC) cells from surgical samples, and in TT cells with the C634W RET mutation (87). In pMTC and TT cells, the proliferation was inhibited significantly (similarly in pMTC cells with/without RET mutation) increasing the apoptosis, by CLM29. Upon the injection of TT cells sc in CD nu/nu mice, neoplastic masses were observed after 20–30 days from xenotransplantation; CLM29 (50 mg/kg/day) significantly decreased tumor growth and microvessel density. These data demonstrated that CLM29 has antineoplastic activity in MTC *in vitro*, and *in vivo*, enabling an eventual future clinical evaluation (87).

Other pyrazolo[3,4-d]pyrimidines (88), such as PP1 (89), PP2 (90), and Si34 (88), have been studied in TC. PP1 pyrazolopyrimidine had an important inhibitory effect on RET kinase (89).

PP2 reduced RET/PTC1-mediated MAPK signaling and inhibited the invasive phenotype and the proliferation of human thyroid carcinoma cells with RET/PTC1 rearrangements (90). PP2 inhibited c-Src and related kinases (91), and for this reason, it was not selective for RET.

Thus, other no direct effects of PP2 mediated *in vivo* by the inhibition of other kinases could not be excluded

This situation was similar for Si34 (88), which inhibits the TK c-Src in 2 human tumor cell lines deriving from MTC, named TT and MZ-CRC-1.

#### Raf Kinase Pathway

Sorafenib (Nexavar^®^) is (a bi-aryl urea) multitargeted TKI, with inhibitor activity against VEGFR-2 and 3, c-Kit, PDGFR, RET/PTC, and Raf kinases (more avidly, C-Raf than B-Raf), and the Raf/Mek/Erk pathway (MAPK pathway); it has been also shown to induce apoptosis through downregulation of Mcl-1 (92, 93).

Sorafenib is approved for the treatment of primary kidney cancer (advanced RCC) and advanced primary liver cancer (HCC).

From the data shown by several phase II trials (62, 63), a multicenter (DECISION trial), double-blind randomized phase III study, that evaluated sorafenib (with respect to placebo), in advanced/metastatic RAI-refractory DTC (with lesions without iodine uptake in a post RAI scan performed during a low iodine diet and with adequate TSH elevation and/or recombinant human TSH stimulation) (64) has been conducted. Patients were randomized 1:1 to receive placebo or sorafenib. The initial group comprises a population of 417 patients (207 treated with sorafenib and 210 with placebo), while the final group was constituted by 416 patients (207 treated with sorafenib and 209 with placebo). The inclusion criteria were: age > 18 years, life expectancy not fewer than 12 weeks, locally advanced or metastatic DTC (PTC, FTC, Hurtle cell, or PDTC) with at least one lesion (measurable by magnetic resonance or computer tomography imaging) and disease progression within 14 months. Other inclusion criteria were a performance status <2 according to Eastern Cooperative Oncology Group, adequate TSH suppression (<0.5 mU/L), absence of renal and liver failure, and adequate bone marrow function. Patients were excluded, if they had yet received any treatment with TKI, monoclonal antibodies against VEGFRs or other targeted agents, chemotherapy or thalidomide. Efficacy and safety of sorafenib have been assessed every 56 days (two cycles) and 28 days (1 cycle) for 8 months and after every 56 days, respectively. At the end of the study, median progression-free survival (PFS) was significantly improved in the patients administered with sorafenib (10.8 months) than the ones with placebo (5.8 months), and it got better in all prespecified clinical and genetic biomarker subgroups, independent from the presence/absence of mutations (64).

A phase III trial, conducted by Bayer, involving 417 patients, is still ongoing (94).

#### VEGF Pathway

Vandetanib (ZD6474; an orally active TKI) has a low molecular weight and a good inhibitory activity of VEGFR-2, and targets VEGFR-3, EGFR, and RET kinases, too (95).

After several trials (65), the international randomized phase III ZETA trial (66) compared ZD6474 (300 mg daily; versus placebo) in 331 patients with MTC: vandetanib prolonged PFS (hazard ratio [HR], 0.46; 95% CI, 0.31–0.69; *P* < 0.001).

FDA approved Vandetanib in April 2011, being the first TKI able to treat adult patients with agressive MTC (66).

One hundred forty-five patients with locally advanced/metastatic DTC, 72 of whom administered with vandetanib (300 mg/daily) and 73 with placebo, improved PFS as shown in a double-blind phase II study (67). TKI-treated patients had a higher PFS (11.1 months) than the ones who received placebo (5.9 months). Partial response (PR) and stable disease (SD) in patients who received TKI were 8% and 57%, while for the ones treated with placebo were 5% and 42%, respectively. The tolerability and safety were in agreement with the ones previously reported by other papers (67).

Various phase I (96) and phase II trials have been performed with (AMG 706) motesanib diphosphate, an ATP-competitive inhibitor of VEGFR-1, -2, and -3, PDGFR, and Kit, administered orally 125 mg/day in patients with metastatic or advanced TC (68, 69).

Motesanib diphosphate was administered to 93 DTC patients [of whom 57 were PTC (61%)] (68): PR was obtained in 14% of the patients, and SD in 35% for 24 weeks (or longer). In 81% of patients, serum Tg diminished with respect to the baseline. Seven patients (8%) had tumor progression and median PFS was 40 weeks. The most frequent adverse events (AEs) were diarrhea (59%), hypertension (56%), asthenia (46%), and weight loss (40%), and the most frequent grade 3 AE was hypertension (25%) (69). Approximately 22% of patients showed primary hypothyroidism.

One hundred eighty-four patients (93 DTC and 91 MTC) were administered in a phase II trial with motesanib (125 mg/day orally) for as far as 48 weeks (97); SD was obtained by 48% of MTC patients for not fewer than 24 weeks. Median PFS was 40 and 48 weeks for DTC and MTC patients, respectively (69).

The second-generation inhibitor of VEGFR-1, -2, and -3, PDGFR, and c-Kit, axitinib (AG-013736) (97, 98), inhibited PDGFR and Kit in cell-based assays more than 10-fold less potently than the other VEGF-TKIs (99).

Sixty patients with advanced TC (30 PTC, 15 FTC, and 11 MTC) were treated with axitinib (5 mg twice daily), in a phase II trial (70). Thirty-eight percent of patients (3 MTC, 12 PTC, and 7 FTC) obtained SD for at least 16 weeks, while 30% (2 MTC, 8 PTC, and 6 FTC) achieved PR. Median PFS was 72.4 weeks (18.1 months). Axitinib had an insignificant effect on KIT, as the authors showed a reduction of soluble VEGFR-2, -3, and Kit of 32%, 35%, and 13% respectively, whereas serum VEGF was 2.8-fold higher (70).

Another phase II trial (71) was conducted with axitinib (5 mg twice daily) in 52 patients with metastatic or locally advanced MTC or DTC. The objective response (OR) rate was 35% (18 PR), and SD was shown in 18 patients for >16 weeks. Median PFS was 16 months, and median overall survival was 27 months. The quality of life was maintained during the treatment. This paper indicates that axitinib could be considered a supplementary option therapy for patients with advanced TC (71).

The multitargeted TKI sunitinib (SU011248) is a selective inhibitor of VEGFR-1, -2, and -3, PDGFR, c-KIT, and RET/PTC subtypes 1 and 3 (100). It has been approved to treat gastrointestinal stromal tumor (GIST), or clear-cell renal carcinoma (101), and it is now investigated in other human cancer types. As AEs, palmar-plantar erythrodysesthesia, fatigue, diarrhea, hypertension, neutropenia, and hypothyroidism have been identified (102).

Sunitinib strongly inhibits the growth of TPC1 cells that have a RET/PTC rearrangement (103).

Another preclinical study (104) indicated that the clinical applications of sunitinib should be directed by genotyping; the study evaluated the different inhibitory mechanisms of this drug against BRAF mutations, or RET/PTC rearrangement in cell lines or orthotopic TC mouse model, showing that it inhibited RET/PTC (but not BRAF) mutated cells (104).

Twenty-eight patients with aggressive DTC and 7 patients with MTC were administered with sunitinib (37.5 mg) on continuous basis in the largest open-label phase II trial (72), showing a complete response (CR) in 3%, PR in 28%, and SD in 46% of patients. The most frequent toxicities were neutropenia (34%), leukopenia (31%), hand/foot syndrome (17%), diarrhea (17%), and fatigue (11%). A decrease in FDG-PET uptake predicted PR or stabilization of the disease (72).

The importance of the therapy with sunitinib in progressive metastatic DTC patients has also been reported by more recently published papers (73).

The oral multiple-receptor kinase inhibitor cabozantinib (XL184) inhibits VEGFR-1 and -2, C-MET, RET, c-Kit, FLT3, and Tie-2 (105, 106).

A phase I trial on cabozantinib was carried out in 37 patients with MTC (74): PR was obtained in 10 (29%) of 35 MTC patients with measurable disease.

Fifteen DTC patients received cabozantinib with 140 mg free base (the same as 175 mg salt form) daily: PR was shown in 8/15 patients (53%), while SD in 6/15 patients (40%) (75).

On the basis of the obtained data, FDA approved cabozantinib for MTC treatment (107).

Also pazopanib (GW786034) is a VEGFR-1, -2, and -3, PDGFR, and c-Kit inhibitor, approved for the treatment of renal cell carcinoma (108). In a phase II trial, 39 patients (37/39 were assessed) with advanced DTC were administered with pazopanib. A PR was achieved in 18 patients (49%), even if no CR was evidenced, with 800 mg/day orally; progressive disease (PD) was seen ultimately in 27 patients (73%). No differences were revealed between PTC and FTC. Tg decreased not <30% in 28/32 patients (88%) (76).

It has been recently demonstrated that the combination of pazopanib with paclitaxel (a microtubule inhibitor) led to a synergistic antitumor activity in ATC cells and xenografts linked to mitotic catastrophe. A pilot clinical translation of pazopanib/paclitaxel treatment showed a lasting effect in a patient with metastatic ATC. The aforementioned data indicated that combining pazopanib and paclitaxel could be effective in the therapeutic approach in ATC (109).

Lenvatinib (E7080) is an oral, multitargeted TKI of VEGFR-1, -2, and -3, FGFR-1, -2, -3, and -4, PDGFR α, RET, and KIT (110). According to the observed data in a phase II study in patients with aggressive TC (77), a phase III study of lenvatinib in Differentiated Cancer of the Thyroid (SELECT) (78) has been conducted. In this phase III study, patients with progressive TC (refractory to iodine-131) were randomly assigned to receive lenvatinib (261 patients; at a daily dose of 24 mg per day in 28-day cycles), or placebo (131 patients). In case of disease progression, patients treated with placebo received open-label lenvatinib. Median PFS was significantly longer (18.3 months) in the lenvatinib group than in the placebo one (3.6 months; *P* < 0.001). All the aforementioned subgroups achieved a PFS associated with lenvatinib. In the lenvatinib group, there were 4 CR and 165 PR, with a response rate of 64.8% versus 1.5% in the placebo group (*P* < 0.001). Median OS was not significantly different in either groups. Patients receiving lenvatinib presented AEs: hypertension (in 68% of the patients), diarrhea (59%), fatigue or asthenia (59%), decreased appetite (50%), decreased weight (46%), and nausea (41%). Because of severe AEs, discontinuation of the study drug happened in 37 patients receiving lenvatinib (14%) and 3 receiving placebo (2%). Drug-related deaths occurred in 6 (out of 20 deaths occurring in the treatment period) patients treated with lenvatinib (78).

#### Vascular Disrupting Drug

Combretastatin A4 phosphate (a microtubule depolymerizing agent) has an effect on tumor vascular networks, leading both severe interruption of tumor blood flow and necrosis to the tumoral mass (111).

In a phase I study, one ATC patient treated with combretastatin showed a CR and was alive 30 months after the treatment (112).

In a phase II study, 18 patients with metastatic ATC, who had not received previously any therapy for advanced disease, were treated with combretastatin intravenously at 45 mg/m^2^ (79). The authors did not observe any ORs and 33% of the subjects had SD, with a reported PFS of 7.4 weeks. The observed AEs were vomiting, mild-to-moderate headache, nausea, and tumor pain.

#### EGFR Pathway

The EGFR inhibitor gefitinib (ZD1839) was employed for the first time in non-small-cell lung cancer (113) and can inhibit with efficacy the ATC proliferation inducing apoptosis *in vitro* (114).

Pennell et al. (80) conducted a phase II trial in 27 patients. Eighteen of 27 patients had an advanced and RAI-refractory DTC and were administered with gefitinib (250 mg/day orally). The more reported AEs and toxicities were rash in 52% of patients, diarrhea (41%), anorexia (11%), nausea (9%). PR was not obtained from any patient; SD was in 48% of patients at 3 months, and in 24% and 12% at 6 and 12 months, respectively. Five among 15 patients (33%) with detectable serum Tg had a strong decrease of it (<90%) for >6 months (80).

As the cytotoxic activity of doxorubicin is increased by the inactivation of EGFR, that also decreases its extrusion; gefitinib and doxorubicin together have been proposed to treat metastatic FTC and ATC (115).

It has been recently shown that there was a PFS of >11 months in a man with metastatic PDTC, showing an EGFR mutation, who responded to the therapy with the selective EGFR TKI erlotinib (59).

#### Histone Deacetylase Inhibitors

US FDA has approved (116) the oral HDAC inhibitor vorinostat (suberoylanilide hydroxamic acid), able to block TC cell growth inducing apoptosis *in vitro* (117), for the treatment of cutaneous T-cell lymphoma. Vorinostat (beginning with the dose of 200 mg b.i.d.) has been evaluated in 19 TC patients (16 DTC and 3 MTC), and none of them had a response (81).

Moreover, owing to the absence of response and a sudden death (grade 5) and a pulmonary embolus (grade 4), a phase II trial about romidepsin in DTC patients was interrupted in 20 patients (118).

#### PPARγ

PPARγ belongs to a superfamily of nuclear hormone receptors (119). Activation of PPARγ isoforms causes both antineoplastic (119) and anti-inflammatory effects (120) in different kinds of mammalian cells. In 2001, a study showed that ligands for PPARγ were able to induce apoptosis and to block the proliferation in human PTC cells (121), to stop distant metastasis of BHP18–21 tumors in nude mice *in vivo* (121), and to induce the process of redifferentiation in TC (122).

For these reasons, Hayashi et al. examined the expression of both PPARγ gene and protein in five human ATC cell lines (123). An elevated level of the PPARγ gene and protein expression was found in five ATC cell lines than in PTC *in vitro*. Cell proliferation was inhibited by PPARγ ligands inducing the process of apoptosis. Moreover, PPARγ ligands downregulated the invasive potential of five ATC cell lines (123).

The *in vitro* biologic effects of the two *PPARγ* agonists ciglitazone and rosiglitazone (RGZ) in ATC cell lines were studied by Aiello et al. (124) showing that RGZ increased the expression of thyroid-specific differentiation markers.

In an *in vitro* study conducted by Marlow et al. (125), it has been shown that the high-affinity PPARγ agonist, RS5444, inducing the cyclin-dependent kinase inhibitor p21, is able to inhibit the proliferation of ATC cells and that the reactivation of suppressed RhoB is a critical step for the growth inhibition of ATC.

Recently, Antonelli et al. have demonstrated *in vitro* that RGZ and pioglitazone (both *PPARγ* agonists) can inhibit the cell growth, and the proliferation in primary cultured cells from human ATC, established from different patients (126).

Moreover, the same group conducted another *in vitro* study in which the results of chemosensitivity tests with PPARγ agonists in primary ATC cells obtained directly from FNA are quite similar than those obtained from surgical biopsies (127, 128).

The aim of a recent phase I study (129), conducted on 15 ATC patients, was to determine the potential effectiveness of paclitaxel and efatutazone (an oral PPARγ agonist) at different doses (seven of them received 0.15 mg, six received 0.3 mg, and two received 0.5 mg of efatutazone). Only one subject, treated with 0.3 mg of efatutazone, had a PR from day 69 to day 175; seven patients had SD. Forty-eight and 68 days were the median times to progression in patients treated with 0.15 and 0.3 mg of efatutazone, and the median survival was 98 versus 138 days, respectively. Grade 3 or greater AEs related to the treatment were exhibited in 10 subjects, in particular, 2 of these were anemia and edema. The combination between efatutazone and paclitaxel resulted in safety and tolerability and had biologic activity (129).

## Conclusion

In spite of the generally good prognosis of TC, ~5% of patients will develop metastatic disease, not responsive to traditional therapies. The knowledge of alterations in different molecular pathways in TC (RET/PTC rearrangements, RET mutations, BRAF mutations, RAS mutations, and VEGFR-2 expression) has permitted the development of new targeted drugs. TKIs are small organic compounds inhibiting TKs auto-phosphorylation and activation and acting on the aforementioned molecular pathways involved in growth, angiogenesis, local, and distant spread of TC. TKIs are emerging as new therapies of aggressive TC, including differentiated TC, MTC, and ATC. Vandetanib and cabozantinib have been approved for the treatment of MTC; sorafenib and lenvatinib have been approved for DTC refractory to RAI. These drugs prolong median PFS, but until now, no significant increase has been observed on overall survival; side effects are common. New efforts are made to find new, more effective, and safe compounds and to personalize the therapy in each TC patient.

## Author Contributions

SMF, PF, UP, GM, EB, SU, PM, and AA gave substantial contribution in the conception and design of the work and in writing the paper. AA revised it critically for important intellectual content. SMF, PF, UP, GM, EB, SU, PM, and AA gave the final approval of the version to be published. SMF, PF, UP, GM, EB, SU, PM, and AA agreed to be accountable for all aspects of the work in ensuring that questions related to the accuracy or integrity of any part of the work are appropriately investigated and resolved.

## Conflict of Interest Statement

The authors declare that the research was conducted in the absence of any commercial or financial relationships that could be construed as a potential conflict of interest.
